# Summary of the Key Concepts on How to Develop a Perinatal Palliative Care Program

**DOI:** 10.3389/fped.2020.596744

**Published:** 2020-12-03

**Authors:** Paola Lago, Maria Elena Cavicchiolo, Francesca Rusalen, Franca Benini

**Affiliations:** ^1^Neonatal Intensive Care Unit, Ca' Foncello Hospital, Treviso, Italy; ^2^Department of Woman and Child Health, Neonatal Intensive Care Unit, University of Padua, Padua, Italy; ^3^Department of Woman and Child Health, Paediatric Pain and Palliative Care Service, University of Padua, Padua, Italy

**Keywords:** perinatal palliative care, limit of viability, life-limiting condition, life-threatening condition, program

## Abstract

**Purpose of review:** The aim of this study is to assess the most significant Perinatal Palliative Care (PnPC) development projects in the literature and summarize the shared key principles.

**Recent findings:** PnPC is a new concept in neonatal intensive care approach. Advancements in perinatal diagnostics and medical technology have changed the landscape of the perinatal world. The threshold of viability continues to decrease, and diagnostic information is available earlier in pregnancy and more rapidly at the bedside; overall outcomes continue to improve. This rapid technological improvement brings ethical debates on the quality of life of patients with life-limiting and life-threatening conditions and the need to involve the family in the decision-making process, according to their wishes and cultural beliefs. Although the Perinatal Hospice concept was developed in the 1980s in the US, the first recommendations on how to develop a PnPC pathway were published in the early 2000s. We considered the most relevant position statements or guidelines on PnPC published in the last two decades. Some of them were more pertinent to pediatrics but still useful for the fundamental concepts and PnPC project's development.

**Summary:** Health care providers and institutions are encouraged to develop PnPC programs, which have the goal of maximizing the quality of life of infants with non-curable conditions. These may generally include the following: a formal prenatal consultation; development of a coordinated birth plan between obstetrician, newborn care, and family; access to other neonatal and pediatric specialties, as needed; comfort palliative care during the prenatal, birth, and postnatal periods; and psychosocial and spiritual support for families, siblings, and staff.

## Key Messages

- PnPC principles and guidelines have been well defined and standardized in the last two decades;
- The candidates to PnPC are well identified: fetuses/infants with life-limiting or threatening conditions that seem incompatible with long-term survival and/or that carries the risk of severe impairment of quality of life as well as prematurity that limits viability;
- Preterm infants born at the limit of viability may seem to be the most challenging candidates because there is often no time to go through the perinatal consultation process and create a birth plan; thus, conflicts could easily emerge between clinicians and staff;
- The key issue is currently the implementation of PnPC into the NICU where we can consider two models: integrative vs. consultative;
- Regardless of the strategy adopted to implement a PnPC pathway in clinical practices, it is mandatory to identify the quality indicators/outcome data and perform regular audits and case debriefing to assure quality improvement;
- Many papers are available on end-of -life care and management of pain and other distressing symptoms;
- Settings for the PnPC program are also important; home discharge and perinatal hospice should be considered in specific cases;
- Staff education is another key rule for a successful implementation of PnPC and should be constantly addressed in the PnPC program.

## Introduction

A Perinatal Palliative Care (PnPC) program aims to optimize quality of life for fetus and infants with life-limiting or life-threatening conditions and their families. The goal of the program is to ensure that infants who are expected to survive only hours, days, or months do not suffer unnecessary pain and discomfort in a family-oriented environment in which parents are allowed to stay with their infant all the time with a grade of involvement based on their wishes and expectations (1).

A dedicated multi-disciplinary team that is comprehensive of all the specialists involved in the perinatal care path should continually reassess all the needs (of the body, mind and spirit) of the newborn and his family while trying to give timely and concrete answers to each of them. This is in accordance with the recent ACOG Committee Opinion #786 entitled Perinatal Palliative Care that is also endorsed by the American Academy of Pediatrics (2, 3).

Perinatal Palliative Care integrates the clinical care of the infant starting from the diagnosis of a life-limiting conditionor from a decision to withholding or withdrawn life support. This also offers an advanced care plan when the transition from intensive care to comfort care is proposed because the best interest of the infants is not prolonging life (2, 3).

Thereafter PnPC may start before birth and continue through comfort palliative care and end-of-life care. After death, there is bereavement care in a continuum of activities to support infant and parents based on their wishes, values, preferences, and spiritual needs. Counseling, debriefing, and emotional support for the staff should also be part of the care path (4).

In all of these steps, it is possible to share an integrated care plan and bundles between the professionals involved to make the process more effective always with respect for the unique individuality of the infants and its family. This study summarizes the development of a PnPC program via a narrative review of the evidence on perinatal management in the world's most industrialized countries.

## Methods

While not adhering to strict PRISMA standards, this review utilized a thorough review of Embase, PubMed, and Google Scholar and the National Bioethics Committed websites via a combination of the following search strings: practice guidelines; AND perinatal palliative care; AND limit of viability. No language restrictions were applied. All potentially relevant titles and abstracts were retrieved and assessed for eligibility.

## Results

We retrieved 34 full text articles from electronic databases and six from National Bioethics Committed websites. We excluded 10 articles as no pertinent with the review. Six papers manually searched were included.

### Evidence From the Literature on PnPC

The application of palliative care concept to the newborn was described for the first time in the United States in 1982 (5). Brian Carter first developed a neonatal end-of-life palliative care protocol in 2002. The protocol was prepared by a 101-member panel using the Delphi research method to build a consensus document. The data collection included both participant input over an 18-month period and synthesis of 16 published and non-published end-of-life protocols developed by international, national, regional, institutional, and parent organizations. While contributors to the project mainly come from the USA, the principles and processes discussed here may be applicable to those in other countries. This work still represents a milestone in the Perinatal Palliative Care program developed all over the word although it focuses mainly on end of life care that is a part of the PnPC program (6).

In 2007, the core standards for Pediatric Palliative Care in Europe were published: IMPaCCT International meeting for Palliative Care in children. Those standards referred to pediatric populations and could also be validated for PnPC in the major points. However, PnPC is a pathway dedicated to fetus and newborns with life-limiting or life-threatening conditions, and thus this standard should be integrated with prenatal consultation and birth plans as well as decision-making on extreme prematurity and the perinatal hospice (7).

In 2013, the American Academy of Pediatrics (AAP) published a Policy Statement on Pediatric Palliative Care and Hospice Commitments, Guidelines, and Recommendations. This team considered children with life-threatening and life-shortening conditions, and they summarized 12 recommendations that should be respected when developing a PnPC program. Briefly, the transversal standards for pediatric palliative care (PPC) include the availability of a dedicated interdisciplinary team that should have sufficient expertise to address all the basic needs of the child and his family (physical, psychological, emotional, practical, and spiritual). The PPC team should provide collaborative integrated multimodal care in relation to the regional pediatrics hospices while maintaining a core level of competency. The PPC team should be consulted frequently if there is a child with life-limiting condition to provide the best and most prompt care possible and should provide communication and decision support, family support, sibling support, and health care professional support. PPC should be a core part of medical school, residency, fellowship, and continuing education curricula. The PPC team should support and engage in research, and the program should have a quality improvement agenda. PPC teams should address ethical issues and engage in relationships with the hospital's ethics committee. The PPC program may have full financial support (8).

The AAP (3), European Foundation for the Care of Newborn Infants (EFCNI) (9), Pediatric Palliative Care Group Australia (10), and national neonatal societies (11–14) have published guidelines and statements specifically regarding PnPC. Table 1 summarizes the most important guidelines.

**Table 1:** Guidelines, position statements and opinion on perinatal palliative care worldwide.

**Country**	**Year**	**Author**	**Methodology**	**Area**	**Topics**
Australia	2018	Paediatric Palliative Care Group Australia	Guidelines	PC	- Family-centered communication and decision-making - Teamwork and coordination of care - Components of care - Use of triggers to recognize children approaching the end of life - Response to concerns - Leadership and governance - Education and training - Supervision and support for interdisciplinary team members - Evaluation, audit and feedback - Systems to support high-quality care
Canada	2016	Limbo et al.	Position statement	PnPC	- Perinatal bereavement - Care coordination - Interprofessional teamwork - Birth and advance care planning - Follow-up
Europe	2007	IMPaCCT: standards for pediatric palliative care in Europe.	Position statement	PPC	- Palliative team - Care coordinator - Pain management - Grief management - Training and education - Euthanasia
Europe	2019	EFCNI (European foundation for the care of newborn infants)	Committee opinion	PnPC	- Decisions of withholding or withdrawing life support - Communication in ethically complex decisions - Rights of infants, parents, and families in difficult decisions - Palliative care
France	2011	French Society of Neonatology and the Working Group on Ethical Issues	Position statement	PnPC	- Euthanasia is forbidden by French law - Physician may lawfully decide to withhold or withdraw a life-sustaining treatment that is unanimously considered unreasonable obstinacy. - Medical decisions must sometimes be taken for a newborn baby whose survival depends on intensive care but whose future quality of life is predicted as poor. - Reasonable treatments that should be started or continued must be distinguished from unreasonable treatments that should be stopped. - This requires assessment of the limit between a quality of life judged as acceptable for the patient and his family and a quality of life judged as unbearable.
Netherlands	2006	Verhagen et al.	Protocol	PnPC	The Groningen Protocol for Euthanasia in Newborns. Requirements that must be fulfilled: - The diagnosis and prognosis must be certain - Hopeless and unbearable suffering must be present - The diagnosis, prognosis, and unbearable suffering must be confirmed by at least one independent doctor - Both parents must give informed consent - The procedure must be performed in accordance with the accepted medical standard
UK	2014	Northern Neonatal Network UK	Guidelines	PPC	- Care before death - Care at the time of death - Care after death - Bereavement support - Staff support and supervision
UK	2015	NANN (National Association of Neonatal Nurse)	Position statement	PnPC	- Palliative care should be offered at any period in which the infant's life may be limited - When a prenatal diagnosis is made, palliative care should be offered prior to delivery. - When continuation of the pregnancy is chosen, planning and decision making for the birth will be present - All care goals should be developed as a team and include the parents. - Family conferences are essential to the providers' understanding of families' needs and their hopes and goals for their infant - An advocate or palliative care provider should be identified prenatally or at delivery/diagnosis for each infant and family in need of palliative services. - Palliative care team - Private home-like environment - Parental preferences - Local hospice - Artificial nutrition - Bereavement care - Support services to all members of the healthcare team
UK	2016	NICE (National Institute for Health and Care Excellence)	Guidelines	PPC	- Advance Care Planning - Emotional and psychological support and interventions - Managing distressing symptoms, such as pain, agitation, seizures or respiratory distress - Hydration and nutrition - Recognizing that a child or young person is likely to die within hours or days - Care and support for parents, carers and healthcare professionals after the death of a child or young person - Care at home
UK	2019	Mancini et al.	Guidelines	PnPC	- Support for Staff: Building Resilience in Nurses - The Importance of Effective Communication on a Neonatal Unit - Spiritual, Cultural and Religious Care for the Baby and Family - Ethical Concepts in Neonatal Palliative Care - Legal Issues in Neonatal Palliative Care - The Principles of Genetics Within Neonatal Palliative Care - Antenatal Care for the Mother and Baby in the Context of Neonatal Palliative Care - Care of Twins, Multiple Births and Support for the Family: A Detailed Background - Care of Twins, Multiple Births and Support for the Family: The Butterfly Project - The Decision-Making Process and the Role of the Neonatal Nurse - Advance Care Planning - Organ and Tissue Donation - Continuing Emotional and Practical Support for the Whole Family - Fundamental Principles of Effective Symptom Management in Neonatal Palliative Care - Collaboration Between Hospices and Neonatal Units - Interdisciplinary Working in Neonatal Palliative Care - Developing Knowledge and Competence in Neonatal Palliative Care Practice - Care after Death
USA USA	2019 2019	ACOG (American College of Obstetricians and Gynecologists) AAP (American Academy of Pediatrics)	Committee opinion Committee opinion	PnPC PnPC	1. Decisions about non-initiation or withdrawal of intensive care should be made by the health care team and the parents of a high-risk infant working together. This approach requires honest and open communication. Ongoing evaluation of the condition and prognosis of the high-risk infant is essential, and the physician, as the spokesperson for the health care team, must convey this information accurately and openly to the parents of the infant 2. Parents should be active participants in the decision making process concerning the treatment of severely ill infants 3. Compassionate basic care to ensure comfort must be provided to all infants, including those for whom intensive care is not being provided 4. The decision to initiate or continue intensive care should be based only on the judgment that the infant will benefit from the intensive care. It is inappropriate for life-prolonging treatment to be continued when the condition is incompatible with life or when the treatment is judged to be harmful, of no benefit, or futile
Germany	2020	PaluTIN group	Committee opinion	PnPC	10-key elements: - Focus on Needs and Hope - Empowering Parenthood - Communication - Evaluating Therapeutic Goals and Treatment Choices Together - Advance Care Planning - Comfort Care Toward the End of Life - Grief Counseling - Spirituality, Religion, and Pastoral Guidance - Support Systems - The Team Duality

*PC, palliative care; PPC, pediatric palliative care; PnPC, perinatal palliative care*.

### Categorization

Guidelines have identified three types of patients for whom PnPC may be anticipated and offered in the newborn period: (a) those born at the threshold of viability; (b) those with birth anomalies that may threaten vital functions, and (c) those for whom intensive care has been appropriately applied but developed an incurable disease (1, 6).

### Multidisciplinary Approach

Most guidelines suggest a multidisciplinary approach involving an interdisciplinary team who can address all the needs of the child and family. It is important to develop an action plan via a multidisciplinary project group with the steps needed to put guidelines into practice at the local level. This may differ from setting to setting. Basically, an effective perinatal palliative care team may include obstetricians, neonatologists, health care providers with expertise in pediatric palliative care, nurses, social workers, mental health professionals, religious counselors, and experts on the child's disease (2, 9, 11, 14). The goal of this team is to provide support and continuity of care throughout pregnancy, delivery, as well as postpartum and neonatal periods. It is important to monitor the data more indicative of the requested changes as in a quality improvement process. This work should review and audit to give evidence of the guideline's implementation.

### Prenatal Diagnosis

Advanced technology can suggest precociously if the fetus is carrying a life-limiting condition. The diagnosis can be made early in pregnancy with invasive or non-invasive prenatal investigations (fetal ultrasound, non-invasive prenatal test, chorionic villous sampling, amniocentesis). The condition may also be suspected and confirmed at birth.

In such cases, antenatal counseling should occur with a multidisciplinary team including at least the gynecologist, the neonatologist, the palliative care team when available, and the consultant with expertise in the child's disease (2, 9, 11, 14). The results of genetic and other diagnostic evaluations, and information about the condition of the fetus and its prognosis should be shared among the group. Different options from termination of pregnancy to palliative comfort care at birth can be presented to the family. A plan of action at birth can be created according to the family's wishes and beliefs (15).

### Birth Plan

A death that is anticipated before birth affords the family the opportunity to work with the health-care team to develop both a birth plan that specifies their desires for how they want the birth to evolve and a palliative care plan. The family's participation in making these plans helps to provide them with a sense of control, and the plan honors their choices for their baby. This is worthwhile in creating a therapeutic alliance for ongoing care. This individualized proposal for delivery and neonatal care could be reviewed during the following consultations until birth. When the birth occurs, the plan should be followed regardless of which health professionals are present at the time (1, 15).

In 2016, the National Institute for Health and Care Excellence (NICE) published guidelines on end-of-life care for infants, children, and young people with the life-limiting conditions: planning and management (16).

NICE's guidelines indicated the modern general principles for developing plans for the care of the child with life-limiting or threatening conditions considering the Advance Care Plan—the preferred tool that considers the preferences and values of parents and caretakers. In the recommendations for research, the NICE committee indicated perinatal palliative care as one of the goals for future research when there is a perinatal diagnosis of life-limiting condition (16).

### Postnatal Diagnosis

There is no opportunity to prepare a birth plan in the case of unanticipated loss, for an unexpected preterm birth, for a life-limiting condition diagnosed postnatally, or in case of a development of an incurable disease.

Limit of viability is a unique condition of PnPC. A systematic review published in Pediatrics in 2015 summarized international recommendations on management of preterm newborn born between 22- and 25 weeks of GA. Although there is a wide variation in clinical practice; the paper identified a gray zone between 23- and 24-weeks GA, general consensus for comfort care at 22 weeks' GA, and agreement for active care after 25 weeks' GA. While this statement may pertain to well-resourced countries, periviability may be referenced in developing or middle-to-poorly resourced countries at gestational ages above 24–25 weeks EGA (17).

Guidelines usually describe a risk-based approach for interventions in the “peri-viable” period by dividing preterm infants into three categories: “*beneficial*” where intervention is generally recommended; “*gray zone*” where interventions follow the decision of parents; and “*futile*” where comfort care is recommended (18–20). This decision cannot be based only on gestational age. For example, across similar time periods, survival rates at 22 weeks' gestation in different countries vary dramatically: from zero in Switzerland and France to as high as 34% in Japan (18, 21). These differences are attributed to either a more active interventionist approach or to underreporting of perinatal deaths. The British Association of Perinatal Medicine (BAPM) proposed a risk-based approach for neonates born <27 gestational weeks based on unmodifiable (i.e., gestational age, fetal growth, sex, plurality) and modifiable risk factors (i.e., prenatal steroids, setting for birth) (22). Some guidelines suggested a more active involvement of families in the decision-making process (2). Others suggested not to resuscitate below some gestational age limit independently of the parents' wishes (17).

In the case of a life-limiting condition diagnosed or confirmed postnatally, it is important to note that the list of diseases could change from country to country and from year to year. As novel therapies emerge and change the disease outcome, management may shift from a palliative care to a more intensive management. For example, in some cases, heart surgery could be offered to children with trisomy 18 (23) and cases of children affected from trisomy 18 who survived longer than a few years have been reported (24). Nevertheless, palliative care teams remain a valuable resource in these settings because they can assist families with decision-making and provide ongoing support since some of these novel therapies are experimental, and the outcomes may be variable.

Similar recommendations are given for children who developed a poor outcome condition after experiencing prolonged intensive care treatment (e.g., severe bronchopulmonary dysplasia or massive necrotizing enterocolitis) or those newborns that cannot improve despite massive medical treatment (Apgar score of 0 at 10 min, refractory pulmonary hypertension, or refractory septic shock). In such cases, guidelines suggest creating a trusting relationship between family and team. The family should be included into the decision-making process without overwhelming them; this can shift the intensive care approach to a palliative one. These transitions may include discontinuation of mechanical ventilator support, artificial circulatory support, and/or artificial nutrition and hydration (2, 9).

### Timing and Setting

Newborns can survive less than a few hours or much depending on the disease. If the diagnosis was made prenatally, then the parents are aware of the child's condition and have a birth plan. The PnPC may be applied in the delivery room. PnPC is generally offered by the neonatologist to guarantee comfort, maintain temperature, and prevent respiratory distress to the newborn (2, 15). Parents should be offered the opportunity to hold and to spend as much time as they wish with their baby in a quiet and private location. After the baby's death, a bereavement care plan should be organized for the family including communicating with parents and creating memories, giving the opportunity to dress the baby and take pictures, and making molds of handprints or footprints—of course while considering the family's spiritual, religious and cultural beliefs (15).

If a life-limiting condition is suspected or confirmed postnatally or there is a shift from intensive care to PnPC, then comfort care may be provided in the neonatal intensive care unit (NICU). Such care includes pain and symptom management, prenatal palliative care consultations, end-of-life care, communication and conflict resolution, collaboration in the care of the medically complex infant, and discharge to hospice where available (2, 9, 14, 16). Amy Kuebelbeck in her website (www.perinatalhospice.org) reported 226 perinatal hospices in the USA and 57 international programs. Perinatal hospice is a family-centered, safe, and quiet place where all of the care is focused on the quality of life of the baby and honoring parent values and wishes; in the case of discharge to home, the focus is on the education of the family on the management of the newborn (25).

### Management

In 2014 Alexandra Mancini et al. “Practical guidance for the management of palliative care on neonatal units” on behalf of the Royal College of Pediatrics and Child Health (26). However, these practical guidelines do not cover the process of reaching the decision to withhold or withdraw life-sustaining treatment and has limited information on decision-making. Other toolkits and practical guidelines are available in this contest. This text focuses on the practical aspect of care including symptoms alleviation and comfort care while providing support to families and staff.

Palliative care has two fundamental concepts: withholding and withdrawing treatment (9, 27). While there may be an emotional difference between not initiating an intervention at all (withholding) and discontinuing it later in the course of care (withdrawing), there is no ethical difference between the two options (28). When an intervention no longer helps to achieve the patient's goals for care or desired quality of life, it is ethically and legally appropriate for physicians to withdraw it and redirect the care. This means that in end-of-life care, the physician can decrease and discontinue the mechanical ventilation and stop any vasoactive drugs, antibiotics, and fluid therapy. Even nutrition, although debated, can be suspended (27–29). The AAP stated that withholding or withdrawing artificial nutrition is acceptable, particularly when it's prolonging the process of dying but children capable to eat and drink safely, who wanted to eat and drink, should be given food and fluids by mouth (28).

This approach is applicable in high resource countries. In a retrospective multicentric study performed in Latin-American NICUs showed a completely different approach in end of life decision making. Withdrawing the support was no existent and usually the baby died with full life support. Preterm babies <27 gestational weeks were not admitted to the NICUs (30).

The European Standards of Care for Newborn Infant Health by EFCNI were published in 2018 and were meant as a benchmark for the development of standards in each individual country (9). They are comprehensive of evidence-based practices and qualitative outcome indicators that will significantly improve health outcomes in preterm infants in Europe and beyond. They developed four standards for key ethical decisions: decision to withhold or withdraw life support; palliative care, communication in ethically complex decisions; and the rights of infants, parents, and families in difficult decisions. They also found that the indicators of meeting the standard were aside from the evidence of a guidelines or formal setup pathway, patient information sheet, training documentations, annual report, or minutes of debriefing. Clearly, parental feedback is a critical issue/factor.

The National Association of Neonatal Nurses (NNAN) published a position statement on palliative and end-of-life care for newborn and infants in 2015 focusing on transport of infant when a redirection of care is chosen as well as nutrition and hydration and on nurse education (31).

Bidegain and Younge proposed two models of PnPC, the “*integrative model*” and the “*consultative model*” (32). The integrative model can be offered early in the pathway of every life-limiting condition or extreme prematurity. Here, the principles of “palliative care” are integrated as core element of the intensive care and after. This perspective could start before birth when a prenatal diagnosis of incurable condition has been made. It could then continue at home or in a hospice context if the child is going to survive longer. The availability of protocols, bundles, and order sets guide clinicians in the process.

In the consultative model, the palliative medicine specialist provides expert advice to the NICU staff on a child with palliative needs. Consultation may focus on same aspect of care as symptom management, consultation, and communication within the primary care team and with families. There is also withdrawal of intensive care and transition to hospice.

Thinking to the implementation of PnPC guidelines, one should consider a combined approach with palliative care principles/resources integrated within the NICU and consultation services available in situations of higher complexity (32).

### Conflicts

In most situations, the care team and family agree about a desire to mitigate pain and suffering including what is in the infant's best interest and how to proceed (2, 9, 22, 26). In some situations however, ethical conflict can arise regarding the provision of potentially inappropriate or no longer beneficial care. This conflict can arise between staff members or between physicians and the family.

Conflicts between parents and staff could be avoided or mitigated involving parents from the starts, allowing time for reflexion (33) or for additional meetings, additional diagnostic tests, and second opinions (34). When the conflict cannot be mitigated, consulting the local ethics committee is suggested (2, 15).

### Milk Donation and Lactation Suppression

Pregnant women with an intrauterine diagnosis of a lethal condition can plan to practice skin to skin contact and breastfeeding with their babies at the time of birth. During the postmortem period, the mothers can be supported if they decide to engage in milk donation (31, 35). Donating breast milk can have a profound influence on how bereaved women make meaning of their experience, and some women described milk donation after perinatal loss as a mechanism to facilitate their progression through grief. However, if a mother decides not to donate milk, then the option of suppressing lactation via pharmacotherapy should be offered (36).

### Following the Death

Although difficult, it is mandatory to give information to the family regarding burial arrangements and final disposition of the infant's body. Verbal and written information about hospital services should be provided to the parents (2, 15). A meeting with the healthcare professionals who actually cared for their baby and parents should be arranged 1–2 months after the loss. During the meeting the clinical report should be provided (15).

### Organ Donation

The issue on organ donation during the neonatal period is controversial. First, it is extremely difficult to ascertain brain death in newborns. It is unclear if giving heparin and intubation to ensure the viability of organs in a baby diagnosed with a life-limiting condition is ethical acceptable. In Italy, organ donation may be considered only term newborns more than 7 days old (>38 gestational weeks) in which brain death is demonstrated (37, 38). In UK organ donation for infants <2 months of age could be performed only if there is a neurological determination of death, which is rarely possible to confirm at this age (39). Ancillary tests, which included electroencephalogram and radionuclide cerebral blood flow study to confirm neurological death could be used in the USA, making the organ donation possible (40).

The strength of our study is that it provides an overview of the most significant aspects of PnPC and aims to provide key principles to create or implement a PnPC program. We recognize some limitation. We did not perform a systematic review but only a narrative review of the most relevant evidence on PnPC in literature and we did not include internal or unpublished guidelines of PnPC that many hospitals might have.

## Discussion

Advancements in perinatal diagnostics and medical technology have changed the landscape of the perinatal world. The threshold of viability continues to decrease, and diagnostic information is available earlier in pregnancy and more rapidly at the bedside. The overall outcomes continue to improve. This rapid technological improvement also brings ethical debates on the quality of life of these patients and the need to involve the family in the decision-making process.

Many professional organizations and scientific societies in industrialized countries promulgate management guidelines for these infants and newborns with poor prognosis pathologies, but the management of these patients, as well as their survival, is very heterogeneous. Survival depends on various factors including social, religious beliefs, law restrictions, and national ethics opinions (41).

In the last two decades, the principles and best practices in a PnPC program were mostly defined along with an overarching palliative care paradigm. However, the literature suggests that there are still many challenging aspects surrounding the implementation of PnPC. These are still lacking in many places were perinatal care are delivered. In our opinion, this is due to several reasons.

First, the key principles of PnPC include advancing care planning, symptom management, and psychosocial support for families and NICU staff even in the neonatal period. Help with the decision-making process and how to manage conflicts and bereavement care are quite definitely defined (2, 9, 15, 41). All of this knowledge is reported as guidelines or evidence-based practices. It is mostly “opinion of the expert” or consensus (based on Delphi methodology) with low levels of evidence. These low levels of evidence may make this research “unconvincing” and may interfere with the implementation of a PnPC.

Second, the outcomes of a “standardized” program and quality improvement initiatives are inconsistently reported. This may due to difficulties in measuring the quality of care/improvement in this setting.

Third, the fact that the PnPC is strongly focused on end-of life care limits the use of PnPC encouraging the misconception that palliative care should be reserved for only end-of-life situations. Rather, it should be considered a comprehensive and coordinated model of care—a curative treatment for fetus/neonates with life-limiting or life-threatening conditions with the goal the quality of life of the infant and family according to their wishes and values.

It is essential to give evidence of rigorous PnPC implementation process as well as evaluation of a wide range of outcome data. Only in this way we can give evidence for successful implementation of PnPC, which is still very low.

The PnPC and its benefits have been endorsed by many institutions and scientific societies (2, 9, 12, 16); however, the PnPC still lags behind other areas of palliative care when it comes to successful implementation of a quality program. Organized programs that include protocols, orders, staff education, and dedicated palliative care teams need to be addressed. The parents' experience is another area to be monitored because this might give the health care professionals insight into the benefits of the best practices in this context.

The continuity of the incurability pathway should be advised because in some situations the infants may survive months and need a different allocation of care. It is also essential that the link between regional pediatric hospice and territorial (primary) care be clear (25). During the life-limiting trajectory, discharge home with hospice support is often in the best interest of the child and their family instead of remaining in the NICU.

PnPC is a new concept in neonatal intensive care approach. Withholding or withdrawing the intensive care does not mean stopping treatments. Rather, it means redirecting active treatment to a different approach (27, 32). PnPC needs education and training of the staff to this new field of care (2, 41). All members of the health care team need to be part of the decision-making process and disagreements between staff or parents and staff needs to be resolved. Opinions from an independent clinical ethics committee may be helpful in resolving conflicts. Organ donation, disposition of the infant remains, and milk donation are elements that should be discussed in a PnPC protocol.

## Conclusion

PnPC is a new field of newborn care. It aims to ameliorate the health status of children with life-limiting conditions. Creating a PnPC program is mandatory to guide physicians and parents through this difficult journey.

## Author Contributions

PL conceptualized and designed the review and drafted the initial manuscript. MC drafted the final version of the manuscript. FB aided in designing the manuscript and contributed to the content. FR reviewed the literature. All authors contributed to the article and approved the submitted version.

## Conflict of Interest

The authors declare that the research was conducted in the absence of any commercial or financial relationships that could be construed as a potential conflict of interest.

## Abbreviations

AAP: American Academy of Pediatrics
ESPNIC: European Society for Paediatric & Neonatal Intensive Care Society
EFCNI: European foundation for the care of newborn infants
IMPaCCT: International meeting for Palliative Care in children
NICE: National Institute for Health and Care Excellence
NICU: neonatal intensive care unit
PnPC: perinatal palliative care
PPC: pediatric palliative care.
